# Gender-based Decision Making in Marketing Channel Choice – Evidence of Maize Supply Chains in Southern Ethiopia

**DOI:** 10.1007/s10745-021-00252-x

**Published:** 2021-09-03

**Authors:** Girma Gezimu Gebre, Hiroshi Isoda, Yuichiro Amekawa, Dil Bahadur Rahut, Hisako Nomura, Takaaki Watanabe

**Affiliations:** 1 Faculty of Environment, Gender and Development Studies, Hawassa University, Hawassa, Ethiopia; 2 Faculty of Agriculture, Kyushu University, Kyushu, Japan; 3 College of International Relations, Ritsumeikan University, Kyoto, Japan; 4 Asian Development Bank Institute, Tokyo, Japan; 5 Department of Agricultural and Resource Economics, Graduate School of Bioresource and Bioenvironmental Sciences, Kyushu University, Fukuoka, Japan; 6 International Maize and Wheat Improvement Center (CIMMYT), El Batan, Mexico

**Keywords:** Maize, Marketing channels, Gender, Decision-makers, Dawuro zone, Ethiopia

## Abstract

We examine factors affecting the choice of marketing channels for maize among male, female, and joint decision-making farm households using data from households in Dawuro zone, southern Ethiopia. Econometric results suggest that female and joint decision-makers are more likely to sell maize to consumers or retailers in the main local market where the maize price is higher than to wholesale merchants directly from the farm. Individual decision-makers (male or female) who grow improved maize varieties are more likely to sell to wholesalers directly from the farm. This may be an indication of the effectiveness of joint decisions over individual decisions related to the market price. We also found that improved maize varieties distributed to farmers in the study area are of poor quality and that there is a lack of modern storage facilities so that farmers have to sell immediately after harvest during the lower price season. Thus, there is a need for policies promoting the distribution of high-quality maize seeds and encouraging investments in the establishment of modern maize storage facilities in the study area.

**Supplementary Information:**

The online version contains supplementary material available at 10.1007/s10745-021-00252-x.

## Introduction

Maize is Ethiopia’s dominant cereal crop in terms of production and number of farms. Averaged over the period 2006 to 2017, 9.5 million smallholder farmers grew maize, over 21% of the total cereal crop production area of the country. Taken together, these farmers produce an annual average of 6.3 million tons, about 30% of total cereal production in Ethiopia (CSA 2015, 2017). Maize accounts for 17–20% of the national per capita calorie intake (Abate *et al.* 2015). The unit cost of calories from maize is the cheapest among all major cereals in Ethiopia, making it the most important cereal crop, particularly for economically less endowed households (Rashid *et al.* 2010; Berhane *et al.* 2011; FAO 2015). Maize is the main staple food for consumers and a critical source of income for smallholder farm households in Ethiopia.

Like many sub-Saharan African countries, maize marketing chains in Ethiopia are relatively long and involve many intermediaries including collectors, wholesalers, or retailers who rarely provide marketing services besides transport and storage. Almost all maize grain reaches consumers without processing. Maize farming households do not receive a reasonable price for their maize harvest because of high transaction costs resulting from poor road access, lack of formal grades and standards, price information asymmetry, high transportation costs, and the presence of intermediaries (Rashid *et al.* 2010; FAO 2015; Abate *et al.* 2015; World Bank 2018), although transaction costs vary across individuals or households according to the type of marketing channel utilized (Hill and Marcella 2014).

Female decision-making farm households face many gender-specific constraints in accessing markets. They tend not to have the same socio-political networks as male decision-making farm households. Men are more likely to be approached by traders or other intermediaries who assume they are the primary decision makers, while women do not have time to search out new market opportunities as they are preoccupied busy with both productive and reproductive household activities. As a result, female-headed households are less successful than male-headed households at accessing new market opportunities (Morrison *et al.* 2007; Barham and Chitemi 2009).

Choice of marketing channel is also determined by the amount of maize being sold (Fafchamps and Hill 2005), which impacts male and female led farm households differentially. There is evidence that female farm households sell smaller quantities at the local market and receive lower prices while male farm households sell bulk quantities and travel to distant markets to secure higher prices (Aregu *et al.* 2011; FAO 2011; Amani 2014; Eerdewijk and Danielsen 2015). Previous studies identify two major reasons for this: first, female-headed households may have fewer productive resources than their male counterparts, so they produce smaller quantities and lack pack animals or money for transport of their produce to distant markets (Fafchamps and Hill 2005; Vigneri and Holmes 2009; Aregu *et al.* 2011; FAO 2011; Amani 2014; Eerdewijk and Danielsen 2015). Second, they often allocate only a small portion of their resources to marketable crops (De Brauw 2015) as they are responsible for family provisioning (Doss 2002).

Smallholder maize farming is a familial system, usually employing one or two household members (Gebre *et al.* 2019). We focus in this study on who makes the marketing decisions in the household. The neoclassical/mainstream economic theory usually regards all the members of the households as having the same preferences. However, agricultural households do not always agree on decisions and women and men do not always have the same preferences (Wilson 1991; Agarwal 1997; Meinzen-Dick *et al.* 2011). Marketing decisions vary among households; in some cases, male and female family members (generally husband and wife) make decisions jointly, while in other cases one or the other make decisions independently (Aregu *et al.* 2011).

We investigate factors affecting maize marketing channel choice by dividing sampled households into three categories for comparison: male, female, and joint decision-making households, to establish any gender differences regarding maize marketing channel choice and the significance this might have on maize producing household well being.

## Materials and Methods

### Study Area, Data Collection, and Sampling Techniques

The principal crops in Dawuro include ensete (*Ensete ventricosum*)*,* teff (*Eragrostis tef*), maize, sorghum, wheat, barley, coffee, beans, peas, spices, vegetables, and fruits. The Dawuro zone has ample potential, but farm productivity is very low because of limitations inherent in traditional means of production, dependence on natural rainfall, and poor market access (Abebe 2014). While both men and women engage in agricultural activities, female-headed farm households are particularly vulnerable because of lack of access to farmland, shortage of farm labour, and whether or not they have draft animals for cultivation.

We collected our data for this study through a household survey, key informant interviews, and focus group discussions conducted in two rounds. In the first round (April-June 2018), we conducted a survey to collect household-level data, and in the second round (June-July in 2019), we conducted key informant interviews and focus group discussions to supplement the survey data.

**Fig. 1:**
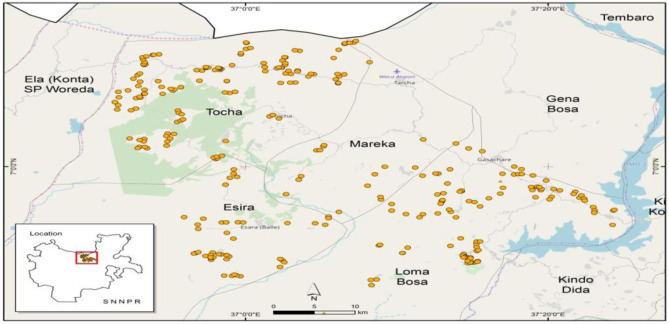
Map of the study area (Dawuro zone) in southern Ethiopia. Source: Authors’ sketch using GPS data (2018)

We used multistage sampling techniques to select smallholder maize farm households for the study. In the first stage, we selected four districts—Loma (including Zisa), Mareka, Esara, and Tocha (Kachi and Tarcha zuriya) based on their maize production and marketing potentials (Fig. 1). In the second stage, we selected 6–8 *kebele*^1^ (peasant associations) from each district where maize is grown as the major staple food for consumption and income. In the third stage, we selected an average of 20 maize growing households to survey from each kebele, for a total of 560 smallholder maize farm households. Since male and female family members work either separately or jointly on the maize farm, we interviewed the person most responsible for production, consumption, and marketing decisions in the household using a semi-structured questionnaire (Gebre *et al.* 2020).

We identified each household as male, female, or joint decision-making based on survey data. All household respondents were asked 20 gender-disaggregated questions (see Appendix). The first 12 pertained to the ownership of farmland and other farm-related assets, maize production decisions, and maize production activities such as variety choice, farm preparation, planting, fertilizer use, weeding, harvesting, and collection. The remaining eight questions related to decision-making about the amount of maize allocated between home consumption and sale, the responsible person in the household for the sale of maize, choice of buyer, price decisions, and utilization of money from the sale of maize. All responses indicated whether decisions were made by men or women, or jointly. Separately, we asked an additional family member supplemental questions, for example, who makes decisions about maize production, consumption, and sale in the market, to ensure we had an accurate description of intra-household gender dynamics. In a few cases, men and women from the same household gave different answers to the same questions in which case we asked them jointly so that they could reach a consensus. Finally, we used principal component analysis^2^ to group all responses into the three household decision-making categories: male, female, or joint.

In each *kebele* we identified key informants such as agricultural experts, community elders, or maize farmers based on information provided by *kebele* level agricultural development agents who closely work with farmers, community elders, and other agricultural experts.We conducted three separate focus group discussions in each sampled district (male, female, and joint decision-making groups) to supplement the household survey data collected. We registered the names, addresses, and identity numbers of surveyed households along with their survey responses.

## Conceptual Framework

Farm households’ choice of marketing channels can be modeled using a random utility framework (Greene 2012) that assumes the choice of a particular maize marketing channel from a set of alternative options is based on its expected utility. Following Greene (2012) and Musara *et al*. (2018), an $i^{th}$ decision maker in the household is faced with *N*(4) market channel choices: own distribution (direct sale to consumers), collectors, wholesalers, or retailers. Then, the utility U of a decision maker *i* making choice *j*, is given as:

1 $${U}_{ij}={B}_{j=k}{X}_{ij}+{\varepsilon }_{ij} \qquad\forall j\epsilon N$$

The vector of variables *X* contains attributes of both the market choice *j* and the decision-maker *i*. A random utility $X_{ij}$ for an individual decision maker choosing a particular alternative is a linear function of a vector of channel-specific parameters ($\beta_{j}$), attributes of individual decision-makers and alternatives ($U_{ij}$), and a stochastic error ($\epsilon_{ij}$ ). If a decision-maker in the household makes choice *j* in particular, then we assume that $U_{ij}$ is the maximum among the *N* alternative utilities. Hence, the probability that the choice *j* is made is denoted as:

2 $${U}_{i(j=k)}>{U}_{i(j\ne k)}\forall k\ne j$$

### Empirical Frameworks

Given that sampled maize farmers in the study areas have more than two alternative market channel choices, we applied the multinomial logit (MNL) model to estimate factors affecting maize marketing channel choice (see, e.g., Deressa *et al.* 2009; Panda and Steerkumar 2012; Arinloye *et al.* 2014; Ndoro *et al.* 2015; Musara *et al.* 2018). It is simpler for computation than the alternatives of multinomial probit, nested logit, and random parameter (mixed) logit models. Following Greene (2012), assuming that the probability that the $i^{th}$ decision-maker in the household chooses the $j^{tk}$ of 4 channels is $p_{ij}$ , the probability that a decision-maker chooses alternative j can takes the form:

3 $${P}_{ij}=\frac{\mathrm{exp}({\beta }_{j}{x}_{i})}{1+\sum\limits^4_{j=1}\mathrm{exp}({\beta }_{j}{x}_{i})}for \;j=1, 2, 3\: \& \:4$$

where $x_{i}$ is a vector of explanatory variables of the $i^{th}$ decision-maker, $\beta_{j}$ is coefficients associated with alternative *j*, and 4 is the number of market channels in the choice set.

The parameter estimates from the multinomial logit regression are difficult to interpret. It is tempting to associate $\beta_{j}$ with the $j^{th}$ outcome, but that could be misleading. It simply gives the direction of explanatory variables on the response (choice) variable; the estimates thus represent neither the actual magnitude of change nor the probabilities associated with each independent variable. By differentiating Eq. (3) with respect to explanatory variables, we identify the marginal effects of individual characteristics on the probabilities, which can be estimated as:

4 $${\delta }_{ij}=\frac{{\partial P}_{ij}}{{\partial x}_{i}}={P}_{ij}\left[{\beta }_{j}-\sum_{j=0}^{4}{P}_{ij}{\beta }_{j}\right]={P}_{ij}\left[{\beta }_{j}-\overline{\beta }\right]$$

Hence, every sub-vector of $\beta$ enters every marginal effect, both through the probabilities and through the weighted average that appears in $\delta_{ij}$ . These values can be computed from the coefficient estimates.

Unbiased and consistent parameter estimates of the MNL model in Eq. (3) require the assumption of independence of irrelevant alternatives (IIA) to hold, which implies that the probability ratio of the decision-maker choosing between two alternative market channels does not depend on the availability or attributes of the other channel choices. This assumption makes MNL somehow restrictive, but it is realistic in situations such as the one under study. The premise of the IIA assumption is the independence between alternatives (i.e., it does not allow correlation between choices) and homoscedasticity of the Eq. (3). Since this assumption is critical, the validity test for IIA is required, for which we used the test developed by Hausman and McFadden (1984), which suggests that if a subset of the choice set is truly irrelevant, then omitting it from the model altogether will not change parameter estimates systematically (Greene 2012). The test result showed no evidence of deviation from the IIA assumption. Hence, there is no need for the trial of other alternative models such as nested logit, random parameter (mixed) logit, or multinomial probit in this study.

### Descriptions of Variables

We identified four major channels through which smallholder farm households in Dawuro zone sold maize during the 2017/18 cropping season: (i) direct sale to consumers in the local market,^3^ (ii) retailers who purchase in the main market,^4^ (iii) wholesalers who purchase in nearby towns,^5^ and (iv) collectors who purchase at the farm gate. Their respective marketing channels are:

i. Producer → Consumer
ii. Producer → Retailer → Consumer
iii. Producer → Wholesaler → Retailer → Consumer
iv. Producer → Collector → Wholesaler → Retailer → Consumer

Hence, the response variable in the empirical estimation is maize farmers’ marketing channel options i to iv. For ease of explanation, here, we present the four patterns of maize supply chain in the order of the increasing numbers of intermediaries from (i) to (iv). However, the priority of maize farm households’ marketing channel choice depends on their utility maximization with consideration for a combination of market margins, amount sold, transaction costs, pests, and disease resistance of maize after harvest, gender of decision-makers, trust of buyers, among others (Table 1 in Appendix).

## Results and Discussion

The results of the pooled sample show that about 38% of the sample households sold their maize directly to consumers in the local market (Table 2 in Appendix). About 19% and 20% sold maize through retailer and wholesaler channels, respectively, while 23% sold through the collectors. About 35% of male decision-making households sold maize through collectors whereas 44% and 39% of female and joint decision-making households sold it directly to consumers in the local market, respectively.

On average, the age of household heads was 42.6 years with the highest average age in households selling maize through wholesalers (43.7 years), followed by those through collectors (43.5 years). In comparison, households that directly sell maize to consumers in the local market owned on average fewer livestock, had a lower rate of improved maize seed application, and allocated a smaller area of their farmland to maize production.

The average age of the household head is highest among joint decision-maker households, particularly those selling maize through collectors, followed by those through consumers (Table 3 in Appendix), and is lowest among female decision-making households. The average years of the household head’s education is higher among female decision-making households than those of male and joint decision-makers. The average number of adult male and adult female family members is highest in male decision-making households, followed by joint decision-making households, and lowest in female decision-making households. The average number of livestock owned is highest in male decision-making households, followed by joint and female decision-making households.

On average, households using improved maize seed, the area of farmland for maize, and the amount of maize sold to the market are higher in male decision-making households than female and joint decision-making farm households. However the average unit price received from maize sale is highest in joint decision-making households while the average unit marketing cost incurred by maize farm households is highest in female decision-making households.

### Econometric Results

To estimate the MNL model first we began by normalizing one category, usually referred to as the base category, in this case ‘consumers in the local market’ since most sampled farm households choose direct sale to consumers in the local market. We then tested for potential endogeneity or any situation in which explanatory variables are correlated with residuals. Other studies have suggested that access to credit service, market information, contact with extension agents, and participation in social events be assumed as endogenous variables in a choice model (e.g., Deressa *et al.* 2009; Mmbando *et al.* 2016). For our test we adopted a two-stage approach that involves the use of predicted values of potentially endogenous variables (Wooldridge 2012). Probit models for access to credit, market information, contact with extension agents, and participation in social events are specified in the first stage. We then used the predicted values of these variables in the second stage of estimating factors affecting farm households’ choice of maize marketing channel. The test failed to reject the null hypothesis, suggesting there is no significant correlation between explanatory variables and residuals. Subsequently, we fitted the Ordinary Least Square model and tested for multicollinearity by using the Variance Inflation Factor (VIF). The VIFs for all the explanatory variables are less than 10 (1.02–1.64), which suggests that there is no serious multicollinearity problem among the explanatory variables included in the model. Finally, we ran the model and tested for the validity of the independence of irrelevant alternative (IIA) assumption by using the Hausman specification test. The test failed to reject the null hypothesis of the independence of the maize marketing channel choice options, suggesting that the MNL specification is appropriate for modeling the maize marketing channel choice of the smallholder maize farm households.

We ran pooled and separate sample models to determine the effect of gender on maize marketing channel choice. In both models, the likelihood ratios indicated by statistics are significant at 1.0% probability, suggesting that both models have a strong explanatory power. The pooled model explains 23.32% of the variation in the market choice among the sampled maize producers. The separate model explains 18.56% of variation in the market choice among male, 48.49% among female, and 39.67% among joint decision-making households, respectively.

As indicated earlier, the coefficient estimates of the MNL model provide only the direction of the effect of the regressor variables on the response variable, i.e., estimated coefficients do not represent the actual magnitude of change or probabilities. Thus, we report and discuss the average marginal effects from the MNL, which helps measure the expected change in the probability of a particular market channel choice being made with respect to a unit change in regressor variables. In all the cases, the estimated coefficients should be compared with the base category of direct sale to consumers in the local market.

In the pooled sample model, the gender of female and joint decision-makers is included to examine the relative positions of an individual or a joint decision-making pair within a farm household while the gender of male decision-makers is considered as a reference group for the analysis. The result indicates that female decision-makers reduce the probability that maize producing households would sell maize to collectors at the farm gate by 9.1% (Table 4 in Appendix). Meanwhile, they had a higher probability of selling maize in the local market by 13.7%.

There are three possible explanations for this result in view of the gender-specific constraints and women’s marketing behaviors in the study area. First, women dominate local market sales by negotiating with buyers who are themselves often local women buying maize for their family consumption. The women peddlers in the local village market have strong social bonds with customers living in the neighborhood. They frequently visit a local market (usually once a week) as they do not have time to travel to the main market located far from the local community, given that they spend significant time each day in obligatory household activities. Second, most women prefer to occasionally sell small quantities in the local market while most men prefer to sell one-shot bulk quantities in more distant markets (Aregu *et al.* 2011). This behavior is mainly linked to the price volatility of maize in the study area as confirmed by agricultural experts, community elders, and farmers; they could be wary of incurring a significant loss by selling in bulk when prices drop. Considering women’s responsibility for family sustainability, they may wish to minimize the risk. This explanation can thus be linked to the generally more risk adverse behavior of women than men (Eckel and Grossman 2008). Lastly, women producers could be less visited by collectors, who may assume that men are primary agricultural producers in the village. This explanation is linked to the notion that men are more likely approached by traders than women for their agricultural products (Barham and Chitemi 2009).

Another result is that joint decision-making households are 13.8% less likely to sell maize to wholesalers in their nearby town. Conversely, they are 13.3% more likely to sell maize in the local market. According to Nyikahadzoi *et al*. (2010), collective marketing reduces the cost of getting the product to markets and helps improve farmers’ bargaining power. The result may thus suggest that joint decision-making households selling their maize products in the local market tend to incur lower transaction costs than do either male or female decision-making households. Another explanation may be related to the tendency for women to play a leading role when they make local marketing decisions. This is exemplified by the following interview narrative provided by a male farmer:

When I sell maize in the local market, I always go there with my wife to help her for transportation and, most importantly, security. I always prefer not to engage in sales activities because most of the buyers there are women and they always charge a lower price for me by saying, ‘you are man, you are the main producer, so do not get involved in this women’s activity’. As a cultural norm, it is no good for us to argue with a woman in the local market. Hence, I let my wife talk to them and she easily negotiates with them for better prices.

His wife in turn confirmed her husband’s view: “I am in charge of selling maize in the local market. However, we [wife and husband] are handling money from the sales together.*”* A further explanation of women’s relatively louder voice in the joint decision-making households could be linked to the price volatility of maize as well as quantitative requirements of the wholesale market. Wholesalers usually require large quantities of produce for purchase although the future price of maize is usually unpredictable for smallholder farmers. Thus, men and women who make joint decisions tend to sell maize in the local market as it caters for much smaller quantities than the wholesale market. In this connection, women who have stronger bonds with customers in the local market are better positioned to take advantage of such relationships for their maize marketing, hence helping to maximize their household economic welfare.

The number of adult male and female family members influences the choice of maize marketing channel. The addition of an adult female in the household decreases the probability by 2.8% that the household would sell maize to retailers in the main market and increases the probability of selling it to consumers in the local market by 2% (cf. Aregu *et al.* 2011). The addition of an adult male in the household increases the probability by 2.6% that they sell maize to collectors at the farm gate; however, they would decrease the probability of selling to consumers in the local market by 1.7% (cf. Amani 2014 for Burkina Faso and Rwanda).

Growing improved maize varieties increases the probability that producers sell maize to retailers in the main market and to collectors at the farm gate by 5.5% each; however, it decreases the probability by 6.3% (as compared to growing traditional maize varieties) that they sell them to wholesalers. These results are linked to the quality of improved maize seeds used by farmers and the storage capacity of traders involved. According to the Ethiopian Seed Association (2014), a lack of quality seed is one of the critical constraints to increasing production and productivity in Ethiopia (see also Gebre *et al.* 2019). On the other hand, the FAO (2015) and World Bank (2018) note that maize traders in Ethiopia face constraints in the capacity of their storage facilities. Maize traders in our study lack capital to invest in large modern maize storage. Compared to other traders, wholesalers are able to store maize for much longer, up to 2 ~ 3 months depending on the market price. Our results indicate that producers are more likely to sell improved maize to retailers or collectors than wholesalers as retailers and collectors sell products immediately after they purchase them. In the study area there are no quality standards nor grades in the maize market, and collectors mix improved maize with traditional maize varieties. Most wholesalers receive maize from collectors, often in such a mixture.

The area of farmland allocated to maize production increases the probability that the farm household sells maize to retailers in the main market rather while it decreases the probability of selling directly to consumers in the local market (cf. Amaya and Alwayng 2011).

The amount of maize households sell influences their choice of marketing channel. Our results indicate that a one-quintal increase in the amount of maize sold increases the probability by 12% that the household sells maize to collectors at the farm gate (with ‘sale to consumers in the local market’ being the base market for comparison). This could be related both to the relatively small amounts required in the local market and poor access to roads and trucks to transport their produce to market. However, it decreases the probability by 1.9% that the household sells maize to wholesalers. An increase in the price of maize increases the probability that farm households sell maize to consumers in the local market while it decreases the probability that they sell maize to wholesalers or collectors.

Since that rational farmers would prefer to sell produce in the market where they can reap the most benefit (Mmbando *et al.* 2016), an increase in the cost of marketing increases the probability that producers sell maize to wholesalers or collectors rather than directly to consumers in the local market (cf. Masuku *et al.* 2001).

The age of the household head increases the probability that female and joint decision-making households sell maize to collectors at the farm gate (Table 5). Older farmers (who are most likely the household head) sell farm produce to a closer market (cf. Amaya and Alwayng 2011; Mmbando *et al.* (2016). As age increases, they lose interest in traveling (even to the local market) and shift to focus on selling produce at the farm gate.

Number of adult females in the male decision-making household is negatively associated with selling to retailers in the main market while positively associated with selling to consumers in the local market. In female decision-making households, number of adult females is positively associated with selling directly to consumers in the local market but negatively associated with selling to wholesalers. In joint decision-making households, an increase in the number of adult females increases the probability by 5.5% that joint decision-makers sell directly to consumers in the local market. The finding might be related to household production capacity. For some agricultural activities such as plowing with oxen and planting, male and female labour is not interchangeable. Plowing with oxen is culturally considered as a male task in the study area (Gebre *et al.* 2020). Thus, given the gendered division of labour for agricultural production, a higher number of adult females in the household (with the number of working age adults in the household held constant) may lead to diminished household farm output. This in turn leads to less marketable produce. Women who prefer to sell smaller quantities are more likely to sell in the local market.

Given the gender division of labour in agriculture, a higher number of adult male family members could provide more household production. In all the three decision-making types of households, an increased number of adult men lead to more sales to collectors. In contrast, an increase in the number of adult males in the male decision-making household decreases the probability of household maize sales to consumers by 3.7% since local market exchanges are dominated by women.

In our sample, the majority of female decision-makers are household heads. The adult males in these households are usually their adult sons may prefer to sell maize to wholesalers and claim the income. Our results show that an adult male added to a female decision-making household increases the probability of household maize sale to consumers or collectors by 4.3% and 5.7%, respectively, whereas the probability of selling maize to wholesalers would decrease by 10.1%.

Similarly, an increase in the number of adult males in joint decision-making households increase the possibility of conflict between men and women selling maize and controlling income. A woman who jointly makes decisions with a man in the household would be unwilling for him to sell maize in distant markets. Our results indicate that in fact they generally agree to sell to collectors at the farm gate and share control over the income from the sale.

Number of livestock owned by male decision-making households increases the probability that they would sell maize to wholesalers in town (cf. Aregu *et al.* 2011).

Planting improved maize varieties decreases the probability that male decision-making households sell to wholesalers in town and increases the probability they sell to collectors at their farm gate. For female and joint decision-making households, growing improved maize variety increases the probability that they sell maize to collectors at the farm gate and retailers in the main market, respectively. Our results also indicate that growing improved varieties results in a decrease in the probability that female and joint decision-makers sell maize to consumers, by 12.8% and 11.5%, respectively but rather sell to collectors at the farm gate. However, collectors in the study area are aware of the farmers’ lack of storage facilities and set a lower price than the market in order to take advantage of the farmers’ need to sell directly after harvest. They also set prices according to their social relationship with the farmer. Male decision-makers with good connections to maize collectors may receive a relatively higher price than female decision-makers. Joint decision-makers are more likely to sell to retailers in the main market where prices are higher.

The area of farmland allocated to maize increases the probability that female and joint decision-making households sell maize to collectors at the farm gate (cf. Amaya and Alwayng 2011) since they usually lack trucks to transport their produce to main/distant markets.

*.*Access to credit services increases the probability that male and joint decision-making households sell maize to collectors at the farm gate. They may receive credit from collectors in advance of maize sales as nationwide evidence suggests (Rashid *et al.* 2010; Abate *et al.* 2015; World Bank 2018). Female decision-makers may fear risks of debt default associated with receiving advance credit from collectors and hence rely on selling directly to consumers in the local market.

## Conclusion and Recommendation

We explore the factors that affect marketing channel choice by comparing three gender-based decision-making household categories: male, female, and joint. Our econometric analyses have four key findings. First, compared with male decision-making households, female decision-making households have a lower probability of selling maize to collectors at the farm gate, and a higher probability of selling to consumers in the local market for three possible reasons: 1) female sellers at the local market have greater bargaining power than men as they prefer to sell to females following customary gender norms; 2) women tend to prefer occasionally selling small quantities in the local market to selling bulk quantities to collectors at the farm gate; 3) females are be visited less often by collectors than males, as collectors, following customary norms, may assume that men are the primary producers or decision makers in the household.

Second, joint decision-making households are less likely to sell maize to wholesalers, and more likely sell to consumers in the local market. In the study area, women tend to be more decisive in joint decision-making than men in choosing the venue for their maize sale between the local market and the wholesale market, because wholesalers mainly engage in bulk purchasing while the future market price of maize is unpredictable for maize producers to ascertain exactly how much they can sell at one time. Accordingly, joint decision-makers tend to sell maize in the local market which caters to the buying of smaller quantities and where female peddlers maintain dominant customary market exchange relationships with other women. Since women in both female and joint decision-making households are largely in charge of maize sales in the local market, there is a need for policies that aim to support women in accessing to a new output market for maize. Moreover, female farm households are less successful than male farm households at searching for accessing new market opportunities for their farm outputs, as women are obliged to engage in both agricultural productive and household maintenance activities.

Third, in all the three decision-making types of households, an increase in the number of adult females in the household increases the probability of selling maize in the local market while an increased number of adult men leads to more sales to collectors at the farm gate. This is related to the gendered division of labour for agricultural production since a larger farm output for sale is associated with more available male labour. Households with more maize output for sale are more likely to sell it to collectors who buy in bulk whereas households with less maize output for sale are more likely to sell it to the local market in smaller amounts.

Fourth, male and female decision-making households that grow improved maize varieties are more likely to sell to collectors at the farm gate due to the quality of improved maize seed and the storage capacity of traders in the study area. Improved maize seeds distributed to the farmers of the study area are susceptible to insect and disease pests and policies and programs should be directed at developing and disseminating insect and disease resistant maize varieties. Further, policies are needed to promote investment in modern storage facilities as maize traders in the study area lack the capital to do so.

## Supplementary Information

Below is the link to the electronic supplementary material.

Supplementary file1 (DOCX 34 KB)

## Footnotes

1 The smallest administrative unit followed by Woreda (district).

2 Principal Component Analysis is a dimensionality-reduction method that is often used to reduce the dimensionality of large data sets, by transforming a large set of variables into a smaller one which still contains most of the information of the large set.

3 ‘Local markets’ are in the vicinity of customers’ households and are smaller than the main market.

4 ‘Main markets’ in small towns and main cities are where traders such as retailers regularly conduct business, and where a larger amount of produce is available.

5 ‘Nearby town’ refers to the main city or a main town of the district where most of the wholesalers live in, such as Gessa Chare, Waka, Essera (Bale and Dali), Tocha (Wara & Aba), and Tarcha towns. Hence, this study considered only one town from each district although there are more than one main markets in each district of the study area.
