# Supplementary material for: Gender-based Decision Making in Marketing Channel Choice – Evidence of Maize Supply Chains in Southern Ethiopia
Source: Hum Ecol Interdiscip J. 2021 Sep 3;49(4):443–51. doi: 10.1007/s10745-021-00252-x (PMC8549917; doi:10.1007/s10745-021-00252-x)
Supplement: Supplementary file 1 — Supplementary file1 (DOCX 34 KB) [file 10745_2021_252_MOESM1_ESM.docx]

Appendix

Questionaries used to cluster households into male, female and joint decision-making groups. Household decision-making and labor division for maize production, consumption and marketing in 2018

|  | Questions | Responses | | |
| --- | --- | --- | --- | --- |
|  |  | Men | Women | Both |
| 1 | Who in the household owns maize farmland? |  |  |  |
| 2 | Who in the household owns farm equipment? |  |  |  |
| 3 | Who in the household owns the crop produce? |  |  |  |
| 4 | Who in the household makes decisions on maize to plant? |  |  |  |
| 5 | Who in the household controls maize farmland? |  |  |  |
| 6 | Who in the household makes decisions on fertilizer use? |  |  |  |
| 7 | Who in the household makes decisions on improved maize seed to use? |  |  |  |
| 8 | Who in the household prepares land for maize production? |  |  |  |
| 9 | Who in the household plants maize on the farmland? |  |  |  |
| 10 | Who in the household weed maize farm? |  |  |  |
| 11 | Who in the household harvests maize? |  |  |  |
| 12 | Who in the household collects the harvested maize to store? |  |  |  |
| 13 | Who in the household made the decision on amount of maize to consume at home? |  |  |  |
| 14 | Who in the household made the decision to sell the maize? |  |  |  |
| 15 | Who in the household decides when to sell? |  |  |  |
| 16 | Who in the household decides when or time of selling? |  |  |  |
| 17 | Who in the household transports the produce to the market? |  |  |  |
| 18 | Who in the household sells maize in the market? |  |  |  |
| 19 | Who in the household makes decisions to choose buyers? |  |  |  |
| 20 | Who in the household makes decision on the use of money obtained from maize sale? |  |  |  |

Table 1 Descriptions of the variables adopted in the empirical model estimate

| **Response Variables** | **Descriptions** | | **Measurement** | | |  |
| --- | --- | --- | --- | --- | --- | --- |
| Market channels | Selling at the farm gate, local market, main market or nearby town | | 1 = Selling to consumers in the local market | | |  |
|  |  |  | 2 = Selling to retailers in the main market | | |  |
|  |  |  | 3 = Selling to wholesalers in the nearby town | | |  |
|  |  |  | 4 = Selling to collectors at the farm gate | | |  |
| **Explanatory Variables** | | | | | |  |
| **Variables** | **Definitions of the Variables** | | | **Measurement** | |  |
| Male decision-makers | | Decision-maker is male in the household | | | 1=yes; 0 otherwise | |
| Female decision-makers | | Decision-maker is female in the household | | | 1=yes; 0 otherwise | |
| Joint decision-makers | | Decision is made jointly by male and female household members | | | 1=yes; 0 otherwise | |
| Age | Age of the household head | | | Year | |  |
| Education | Educational level of the household head (total years of schooling) | | | Year | |  |
| Children | Number of children in the household (age $\leq$ 15) | | | Person | |  |
| Adult female | Number of the adult female in the household (age > 15) | | | Person | |  |
| Adult male | Number of the adult male in the household (age > 15) | | | Person | |  |
| Livestock | Total number of livestock owned by the household | | | Tropical Livestock Unit (TLU) | |  |
| Improved maize | Improved maize varieties used by farm the household | | | 1 = yes; 0 otherwise | |  |
| Maize land | The size of farmland allocated to maize production | | | Hectare | |  |
| Maize sold | Amount of maize sold by household in 2018 | | | Quintal (100 kg) | |  |
| Price of maize | Price of the maize sold in 2018 | | | ETB (Ethiopian Birr) per quintal^[[1]](#footnote-1)^ | |  |
| Marketing costs | Total marketing cost of maize per quintals for channel for the households | | | Ethiopia birr (ETB)^[[2]](#footnote-2)^ | |  |
| Distance | Distance to the nearest maize market | | | Kilometer | |  |
| Credit | Access to credit services | | | 1= yes; 0 otherwise | |  |
| Extension | Contact with extension agent | | | 1= yes; 0 otherwise | |  |
| Social events | Participation in social events. | | | 1= yes; 0 otherwise | |  |
| Information | Access to information on maize market | | | 1= yes; 0 otherwise | |  |

Table 2 The mean values of the socio-economic characteristics of pooled sample maize producers (N =560)

|  | Marketing channels | | | | Total |
| --- | --- | --- | --- | --- | --- |
| Variables | Consumer | Retailer | Wholesaler | Collector |  |
| Male decision-makers (N=240) | 0.24 | 0.18 | 0.23 | 0.35 | 0.43 |
| Female decision-makers (N=118) | 0.44 | 0.21 | 0.19 | 0.16 | 0.21 |
| Joint decision-makers (N =202) | 0.39 | 0.22 | 0.15 | 0.24 | 0.36 |
| Total | 0.38 | 0.19 | 0.20 | 0.23 | 100 |
| Age | 41.88 | 41.68 | 43.49 | 43.70 | 42.61 |
| Education | 3.06 | 3.86 | 3.41 | 3.84 | 3.43 |
| Children | 2.13 | 2.11 | 2.07 | 2.14 | 2.12 |
| Adult female | 2.18 | 1.77 | 1.79 | 1.80 | 1.88 |
| Adult male | 1.99 | 2.13 | 2.15 | 2.35 | 2.18 |
| Livestock | 5.57 | 6.30 | 6.23 | 6.44 | 6.03 |
| Improved maize | 0.55 | 0.70 | 0.63 | 0.78 | 0.65 |
| Maize land | 0.67 | 0.90 | 0.91 | 0.93 | 0.82 |
| Maize sold | 11.26 | 9.20 | 8.60 | 20.20 | 12.31 |
| Price of maize | 782 | 780 | 755 | 745 | 765.50 |
| Marketing costs | 36.14 | 85.10 | 117.30 | 187.73 | 106 |
| Distance | 11.05 | 10.80 | 12.21 | 10.80 | 10.88 |
| Credit | 0.32 | 0.39 | 0.40 | 0.45 | 0.38 |
| Extension | 0.77 | 0.74 | 0.74 | 0.87 | 0.78 |
| Social events | 0.70 | 0.62 | 0.60 | 0.68 | 0.66 |
| Information | 0.70 | 0.74 | 0.76 | 0.75 | 0.73 |

Table 3 The mean values of the socio-economic characteristics by gender of decision-makers in the household

|  | Male (N=240) | | | | | Female (N=118) | | | | | Joint (N =202) | | | | |
| --- | --- | --- | --- | --- | --- | --- | --- | --- | --- | --- | --- | --- | --- | --- | --- |
|  | Marketing channels | | | | | Marketing channels | | | | | Marketing channels | | | | |
| Variables | Consumer | Retailer | Wholesale | Collector | **Total** | Consumer | Retailer | Wholesaler | Collector | **Total** | Consumer | Retailer | Wholesaler | Collector | **Total** |
| Age | 42.20 | 41.83 | 43.24 | 42.14 | 42.40 | 40.10 | 41.12 | 41.33 | 43.60 | 41.20 | 43.31 | 41.43 | 41.61 | 45.26 | 43.50 |
| Education | 2.85 | 3.15 | 4.20 | 3.76 | 3.10 | 3.02 | 4.02 | 3.00 | 4.27 | 3.63 | 2.92 | 3.27 | 3.20 | 2.90 | 3.02 |
| Children | 1.72 | 1.65 | 1.60 | 1.96 | 2.32 | 2.44 | 2.95 | 2.46 | 2.27 | 2.11 | 2.33 | 2.20 | 2.70 | 2.28 | 2.00 |
| Adult female | 2.08 | 1.74 | 1.77 | 2.07 | 2.06 | 1.95 | 1.82 | 1.67 | 1.89 | 1.89 | 2.00 | 1.71 | 1.82 | 1.91 | 1.95 |
| Adult male | 2.10 | 2.37 | 2.30 | 2.44 | 2.16 | 2.22 | 2.18 | 1.83 | 2.36 | 2.00 | 1.68 | 1.81 | 2.00 | 2.10 | 2.05 |
| Livestock | 5.45 | 5.27 | 7.78 | 5.90 | 6.50 | 6.00 | 5.90 | 4.98 | 5.37 | 5.80 | 5.43 | 5.68 | 6.90 | 6.70 | 6.00 |
| Improved maize | 0.56 | 0.66 | 0.45 | 0.83 | 0.68 | 0.54 | 0.72 | 0.62 | 0.75 | 0.63 | 0.52 | 0.75 | 0.72 | 0.73 | 0.65 |
| Maize land | 0.75 | 0.80 | 0.96 | 0.98 | 0.96 | 0.73 | 0.92 | 0.85 | 0.94 | 0.90 | 0.67 | 0.84 | 0.86 | 0.88 | 0.78 |
| Maize sold | 6.40 | 10.30 | 16.00 | 27.10 | 14.93 | 14.38 | 11.67 | 9.70 | 12.43 | 11.07 | 14.09 | 10.77 | 7.03 | 18.44 | 12.76 |
| Price of maize | 802 | 815 | 776 | 768 | 790 | 720 | 732 | 690 | 687 | 707 | 820 | 835 | 787 | 777 | 804 |
| Marketing costs | 35.30 | 84.25 | 117 | 187.8 | 106 | 41.37 | 90.33 | 118.77 | 187.87 | 109 | 34.44 | 83.40 | 116.34 | 187.11 | 105 |
| Distance | 9.16 | 9.02 | 10.45 | 10.50 | 10.63 | 12.21 | 10.02 | 9.85 | 10.70 | 11.34 | 11.50 | 9.60 | 12.52 | 10.20 | 10.90 |
| Credit | 0.27 | 0.37 | 0.48 | 0.50 | 0.36 | 0.36 | 0.27 | 0.38 | 0.27 | 0.35 | 0.28 | 0.28 | 0.35 | 0.37 | 0.33 |
| Extension | 0.71 | 0.72 | 0.74 | 0.86 | 0.80 | 0.86 | 0.72 | 0.81 | 0.74 | 0.76 | 0.77 | 0.76 | 0.58 | 0.89 | 0.78 |
| Social events | 0.78 | 0.70 | 0.63 | 0.68 | 0.70 | 0.68 | 0.69 | 0.64 | 0.63 | 0.63 | 0.68 | 0.57 | 0.64 | 0.73 | 0.65 |
| Information | 0.68 | 0.70 | 0.78 | 0.72 | 0.70 | 0.70 | 0.62 | 0.73 | 0.71 | 0.67 | 0.72 | 0.78 | 0.72 | 0.78 | 0.75 |

Source: Own survey result (2018)

Table 4 Average Marginal Effects through MNL on factors affecting maize market channel choice by farm households

| Variables | Consumer | Retailers | Wholesalers | Collectors |
| --- | --- | --- | --- | --- |
|  | dy/dx | dy/dx | dy/dx | dy/dx |
| Female decision-makers | 0.137*** (0.046) | 0.012 (0.043) | -0.058 (0.046) | -0.091** (0.042) |
| Joint decision-makers | 0.133*** (0.043) | 0.045 (0.041) | -0.138*** (0.038) | -0.040 (0.041) |
| Age | -0.001 (0.002) | -0.001 (0.002) | 0.001 (0.002) | 0.002 (0.002) |
| Education | -0.001 (0.005) | 0.003 (0.004) | 0.003 (0.004) | 0.001 (0.004) |
| Children | -0.009 (0.011) | -0.006 (0.011) | 0.001 (0.011) | 0.014 (0.011) |
| Adult female | 0.002** (0.018) | -0.028** (0.018) | 0.012 (0.017) | 0.015 (0.017) |
| Adult male | -0.017** (0.014) | 0.007 (0.013) | 0.016 (0.013) | 0.026** (0.013) |
| Livestock | -0.003 (0.004) | 0.001 (0.004) | 0.000 (0.004) | 0.002 (0.004) |
| Improved maize | -0.047 (0.037) | 0.055** (0.037) | -0.063* (0.035) | 0.055*** (0.038) |
| Maize land | -0.046** (0.032) | 0.046** (0.027) | 0.005 (0.027) | 0.004 (0.027) |
| Maize sold | -0.052* (0.006) | -0.013 (0.003) | -0.019** (0.002) | 0.120*** (0.022) |
| Price of maize | 0.001** (0.002) | 0.002 (0.003) | -0.001** (0.001) | -0.002*** (0.004) |
| Marketing costs | -0.002*** (0.003) | -0.003 (0.003) | 0.004** (0.002) | 0.003*** (0.003) |
| Distance | -0.002 (0.003) | -0.006 (0.003) | 0.007 0.003) | 0.001 (0.003) |
| Credit | -0.059** (0.037) | 0.012 (0.035) | 0.013 (0.034) | 0.033 (0.035) |
| Extension | 0.051 (0.042) | -0.053 (0.039) | -0.081 (0.038) | 0.084 (0.044) |
| Social events | 0.041 (0.036) | -0.049 (0.033) | -0.014 (0.032) | 0.022 (0.034) |
| Information | -0.010 (0.041) | 0.032 (0.039) | 0.041 (0.038) | 0.019 (0.039) |
| **Diagnosis** |  | |  |  |
| Base category | Consumers | |  |  |
| Log likelihood | -575.827 | |  |  |
| Prob > chi2 | 0.000 | |  |  |
| R^2^ | 0.2332 | |  |  |
| N | 560 | |  |  |

***, ** and * denote level of significance at 1%, 5% and 10%, respectively. Standard Errors are given in parentheses.

Table 5 Average Marginal effects through MNL on factors affecting maize market channel choice by gender of decision-makers in the household

|  |  | Male decision-makers | |  |  | Female decision-makers | |  |  | Joint decision-makers | |  |
| --- | --- | --- | --- | --- | --- | --- | --- | --- | --- | --- | --- | --- |
| Variables | Consumer | Retailers | Wholesale | Collectors | Consumer | Retailers | Wholesaler | Collectors | Consumer | Retailers | Wholesaler | Collectors |
|  | dy/dx | dy/dx | dy/dx | dy/dx | dy/dx | dy/dx | dy/dx | dy/dx | dy/dx | dy/dx | dy/dx | dy/dx |
| Age | -0.002 (0.003) | -0.002 (0.003) | 0.003 (0.003) | 0.001 (0.003) | 0.001 (0.003) | -0.003 (0.004) | 0.006 (0.003) | 0.004** (0.003) | 0.001 (0.003) | -0.003 (0.003) | -0.001 (0.002) | 0.005** (0.003) |
| Education | -0.002 (0.008) | 0.000 (0.007) | 0.006 (0.007) | 0.004 (0.007) | -0.003 (0.010) | 0.012 (0.011) | -0.023 (0.010) | 0.009 (0.010) | -0.006 (0.007) | 0.001 (0.007) | 0.014 (0.007) | -0.009 (0.007) |
| Children | -0.011 (0.020) | -0.009 (0.018) | -0.010 (0.018) | 0.030 (0.018) | -0.015 (0.020) | 0.041 (0.024) | -0.023 (0.021) | -0.003 (0.023) | 0.001 (0.017) | -0.022 (0.017) | 0.004 (0.015) | -0.017 (0.016) |
| Adult female | 0.015* (0.029) | -0.041** (0.028) | -0.013 (0.027) | 0.042 (0.027) | 0.010** (0.033) | -0.060 (0.041) | -0.092** 0.042 | -0.042 0.039 | 0.055** 0.031 | -0.038 0.032 | -0.021 (0.022) | -0.038 (0.026) |
| Adult male | -0.037** (0.022) | 0.021 (0.019) | 0.003 (0.020) | 0.019** (0.021) | 0.043* (0.029) | 0.000 (0.027) | -0.101*** (0.033) | 0.057** (0.025) | -0.037 (0.025) | 0.005 (0.026) | 0.007 (0.018) | 0.035* (0.020) |
| Livestock | -0.001 (0.007) | -0.004 (0.008) | 0.008** (0.006) | 0.005 (0.008) | 0.011 (0.012) | 0.001 (0.013) | -0.014 (0.013) | -0.002 (0.013) | -0.002 (0.008) | 0.002 (0.008) | 0.004 (0.006) | 0.001 (0.007) |
| Improved maize | -0.012 (0.057) | 0.018 (0.051) | -0.084** (0.055) | 0.078** (0.057) | -0.128** (0.098) | 0.120 (0.114) | -0.089 0.097 | 0.097* 0.108 | -0.115** 0.064 | 0.135* (0.072) | 0.033 (0.060) | 0.052 (0.061) |
| Maize land | -0.016 (0.054) | 0.031 (0.048) | 0.025 (0.049) | 0.022 (0.050) | -0.123** (0.061) | 0.059 (0.058) | -0.016 (0.054) | 0.086** (0.062) | -0.097** (0.049) | 0.070 (0.044) | 0.020 (0.033) | 0.007* (0.038) |
| Maize sold | -0.034*** (0.008) | 0.002 (0.001) | 0.015*** (0.003) | 0.016*** (0.003) | 0.076*** (0.012) | 0.028 (0.008) | -0.030*** 0.007 | -0.018** 0.005 | 0.058*** (0.010) | -0.018 (0.007) | -0.017*** (0.003) | 0.023*** (0.004) |
| Price of maize | 0.001 (0.000) | 0.003** (0.002) | -0.001 (0.002) | -0.003 (0.001) | 0.001** (0.001) | 0.017 (0.007) | -0.000** (0.000) | -0.000** (0.000) | 0.002** (0.003) | 0.002* (0.004) | -0.001 (0.001) | -0.001** (0.000) |
| Marketing costs | -0.003*** (0.004) | -0.001 (0.004) | 0.001** (0.004) | 0.003*** (0.004) | -0.005** (0.006) | -0.017 (0.007) | 0.012** (0.007) | 0.005*** (0.007) | -0.004** (0.006) | 0.001 (0.006) | 0.005** 0.004)( | 0.002** (0.005) |
| Distance | -0.006 (0.006) | -0.006 (0.006) | 0.005 (0.006) | 0.007 (0.006) | -0.003 (0.010) | 0.010 (0.011) | -0.012 (0.010) | 0.004 (0.010) | 0.003 (0.004) | -0.010 (0.004) | 0.007 (0.003) | 0.000 (0.004) |
| Credit | -0.063 (0.064) | 0.022 (0.057) | 0.039 (0.058) | 0.001** (0.058) | -0.020 (0.072) | 0.043 (0.079) | -0.001 (0.075) | -0.021 (0.078) | -0.095* (0.054) | -0.011 (0.057) | 0.001 (0.050) | 0.105** (0.057) |
| Extension | -0.029 (0.067) | 0.014 (0.062) | 0.077 (0.065) | 0.119 (0.073) | 0.063 (0.085) | -0.143 (0.090) | 0.169 (0.097) | -0.089 (0.088) | 0.100 (0.063) | -0.023 (0.065) | -0.137 (0.050) | 0.060 (0.064) |
| Social events | 0.111 (0.056) | 0.021 (0.050) | -0.093 (0.053) | 0.003 (0.054) | -0.021 (0.071) | -0.018 (0.076) | 0.006 (0.074) | 0.032 (0.072) | -0.034 (0.058) | -0.081 (0.054) | 0.030 (0.048) | 0.085 (0.056) |
| Information | -0.074 (0.055) | -0.008 (0.051) | 0.110** (0.057) | -0.027 (0.056) | 0.246 (0.103) | -0.193 (0.125) | 0.017 (0.116) | -0.070 (0.122) | 0.041 (0.075) | -0.063 (0.080) | 0.018 (0.065) | 0.005 (0.074) |
| **Diagnosis** |  | | | |  | | | |  | | | |
| Base category | Consumers | | | | Consumers | | | | Consumers | | | |
| Log likelihood | -266.281 | | | | -79.870 | | | | -159.217 | | | |
| Prob > chi2 | 0.0000 | | | | 0.0000 | | | | 0.0000 | | | |
| Pseudo R2 | 0.1856 | | | | 0.4849 | | | | 0.3967 | | | |
| N | 240 | | | | 118 | | | | 202 | | | |

****, ** and * denote level of significance at 1%, 5% and 10%, respectively. Standard Errors are given in parentheses*

1. Quintal is a unit of weight equal to 100 kg. [↑](#footnote-ref-1)
2. One ETB (Ethiopian Birr or currency) was equal to USD 0.036 during the data collection period*.* [↑](#footnote-ref-2)
